# Photocurable Bioinks for the 3D Pharming of Combination Therapies

**DOI:** 10.3390/polym10121372

**Published:** 2018-12-11

**Authors:** Giovanny F. Acosta-Vélez, Chase S. Linsley, Timothy Z. Zhu, Willie Wu, Benjamin M. Wu

**Affiliations:** 1 Department of Bioengineering, University of California, Los Angeles, 420 Westwood Plaza, Room 5121, Engineering V, 951600 Los Angeles, CA 90095, USA; gacosta3@g.ucla.edu (G.F.A.-V.); clinsle@g.ucla.edu (C.S.L.); timmy90109@gmail.com (T.Z.Z.); williewu@ucla.edu (W.W.); 2 Division of Advanced Prosthodontics and the Weintraub Center for Reconstructive Biotechnology, University of California, 951600 Los Angeles, CA 90095, USA

**Keywords:** 3D printing, pharmaceutical tablets, poly(ethylene glycol), hyaluronic acid, photopolymerization, inkjet printing

## Abstract

Combination therapies mediate drug synergy to improve treatment efficacy and convenience, leading to higher levels of compliance. However, there are challenges with their manufacturing as well as reduced flexibility in dosing options. This study reports on the design and characterization of a polypill fabricated through the combination of material jetting and binder jetting for the treatment of hypertension. The drugs lisinopril and spironolactone were loaded into hydrophilic hyaluronic acid and hydrophobic poly(ethylene glycol) (PEG) photocurable bioinks, respectively, and dispensed through a piezoelectric nozzle onto a blank preform tablet composed of two attachable compartments fabricated via binder jetting 3D printing. The bioinks were photopolymerized and their mechanical properties were assessed via Instron testing. Scanning electron microscopy (SEM) was performed to indicate morphological analysis. The polypill was ensembled and drug release analysis was performed. Droplet formation of bioinks loaded with hydrophilic and hydrophobic active pharmaceutical ingredients (APIs) was achieved and subsequently polymerized after a controlled dosage was dispensed onto preform tablet compartments. High-performance liquid chromatography (HPLC) analysis showed sustained release profiles for each of the loaded compounds. This study confirms the potential of material jetting in conjunction with binder jetting techniques (powder-bed 3D printing), for the production of combination therapy oral dosage forms involving both hydrophilic and hydrophobic drugs.

## 1. Introduction

Personalized medicine is becoming a reality. For instance, most hearing aids are now custom-fit to each user’s ear canal [1] and the manufacturing process for Invisalign^TM^ aligners and retainers utilizes 3D printing [2]. Additionally, custom bioresorbable tracheal splints fabricated using laser-based 3D printing were successfully used to treat children with tracheobronchomalacia [3]. Recently, interest in applying 3D printing to the manufacturing of pharmaceutical tablets—known as 3D pharming [4]—has been gaining traction since it can allow for on-demand manufacturing of personalized pharmaceutical dosage forms [5,6]. It also allows for the creation of dosage forms with increased complexity, including the manufacturing of oral dosages containing multiple active pharmaceutical ingredients (APIs), which aim to enhance patient compliance by reducing the number of pills required on a daily basis [7]. Additionally, several medical conditions utilize combination therapies to improve treatment efficacy, such as hypertension, HIV infection, depression, type 2 diabetes, and cancer [8,9,10,11,12,13,14,15,16,17].

There are several challenges to manufacturing dosage forms containing multiple APIs as well as dosing constraints that impact treatment efficacy. Currently, a few fixed dose combinations of varying strength are created for any given combination therapy. However, patients often require dose adjustments to the extent that a tablet with multiple APIs is not a suitable form factor [18]. Additionally, there are challenges to manufacturing multiple-API dosage forms [19]. For instance, a common challenge is designing a single dosage form that combines two chemically incompatible APIs that have to be kept separated. This can result in higher manufacturing costs since a more complex dosage form has to be produced. Three-dimensional pharming is uniquely positioned to overcome these obstacles. In fact, techniques such as fused deposition modeling [20] and extrusion printing at room temperature [21,22] have demonstrated feasibility in the manufacturing of pharmaceutical tablets with multiple APIs. Material jetting technologies, however, allow for compositional control at a voxel level, thereby enabling precise dosage control that is tailorable to each individual patient. Additionally, material can be dispensed at room temperature, which is compatible with thermally labile APIs.

The focus of this work was to demonstrate that 3D printing and the development of photocurable formulations can be combined to fabricate pharmaceutical tablets with controlled dosages of both hydrophilic and hydrophobic APIs, and act as a potential carrier for personalized medicine treatments. Previously, this lab engineered a hyaluronic acid-based photocurable bioink for the 3D pharming of hydrophilic compounds via material jetting [23]. Additionally, this lab engineered a poly(ethylene glycol) diacrylate-based bioink capable of loading hydrophobic drugs [24]. In this paper, both bioinks are used to manufacture a tablet with multiple APIs used to treat hypertension. Specifically, the APIs lisinopril and spironolactone were chosen as model drugs for this combination therapy. Lisinopril, an angiotensin-converting enzyme inhibitor (ACEI) for the treatment of hypertension [25,26], was chosen due to its hydrophilicity, and spironolactone, a potassium-sparing diuretic for the treatment of hypertension and congestive heart failure [27], was selected because of its hydrophobicity. The formulated bioinks were dispensed through a piezoelectric nozzle at room temperature into a blank preform tablet featuring two compartments, one for each formulation. Compartmentalizing the preform tablet was required in order to expose each formulation, separately, to the required light exposure times resulting in optimal mechanical properties and release profiles. The preform tablet parts were manufactured by binder jetting process, using calcium sulfate powder as the excipient. The drug-loaded bioinks were subsequently photopolymerized, and the preform tablet parts were assembled to finalize the pharmaceutical dosage form. This tablet produced a sustained drug release profile for both APIs and demonstrated that this 3D pharming approach could successfully fabricate oral combination therapies at room temperature, with quick manufacturing times and controlled dosages.

## 2. Materials and Methods

### 2.1. Hydrophilic Photocurable Bioink Preparation

Hyaluronic acid norbornene (HANB) was synthesized as previously described [23]. Briefly, hyaluronic acid (HA) (60 kDa·MW) (Genzyme Corporation, Cambridge, MA, USA) was modified with hydrazide groups through a reaction with adipic acid dihydrazide (ADH) in the presence of 1-ethyl-3-(dimethylaminopropyl) carbodiimide hydrochloride (EDC). The product was dialyzed for 3 days against deionized water (DI) water (Fisherbrand regenerated cellulose, MWCO 12,000–14,000 Da, Houston, TX, USA), frozen, and lyophilized. On a second reaction, the HA functionalized with hydrazide groups (HA–ADH) was reacted cis-5-norbornene-*endo*-2,3-dicarboxylic anhydride (Sigma–Aldrich, St. Louis, MO, USA), resulting in norbornene-functionalized HA. The product was dialyzed against DI water for 3 days, filtered, lyophilized, and stored at −20 °C. Norbornene-functionalized HA was characterized by proton nuclear magnetic resonance spectroscopy (^1^H NMR) on a Bruker AV300 broad band FT NMR Spectrometer (Billerica, MA, USA). The degree of modification obtained was ~50%. 

Following its synthesis, HANB was dissolved in PBS and mixed with poly(ethylene glycol) dithiol (1500 Da, PEGDT) at a crosslinking ratio (ratio of thiol groups to norbornene groups, *r*_ratio_) of 0.6. Norbornene-functionalized HA was added at a weight percent (*W*_HANB_) of 3%. Eosin Y was added as a photoinitiator and poly(ethylene glycol) (PEG, 200 Da) was added to optimize the viscosity of the formulation for droplet formation. Each of these two components constituted 10% v/v of the bioink. Lisinopril dihydrate (Fisher Scientific, Pittsburgh, PA, USA) was added at a concentration of 40 mg/mL. All chemicals were purchased from Sigma–Aldrich unless otherwise stated. 

### 2.2. Hydrophobic Photocurable Bioink Preparation

The hydrophobic bioink was formulated as previously described [24], with minor modifications. Briefly, the bioink was composed of 30% poly(ethylene glycol) diacrylate (PEGDA, 250 Da), 50% PEG200, and 20% ethanol. Eosin Y (1.0 mM) and mPEG-amine (0.05 M) (350 Da, Creative PEG Works, Chapel Hill, NC, USA) were added as photoinitiator and co-initiator, respectively. Spironolactone was added at a concentration of 20 mg/mL. All chemicals were purchased from Sigma–Aldrich unless otherwise stated.

### 2.3. Bioinks Gelation and Mechanical Properties

The tensile strength of polymerized gels was analyzed to characterize the mechanical properties of the bioinks. One-mL syringes (BD & Co., Franklin Lakes, NJ, USA), modified by eliminating their tips, were loaded with 50 µL of bioink. Gels were formed with hydrophilic and hydrophobic bioinks by exposing them to visible light at an intensity of 120 mW/cm^2^ for a period of 2 min and 1 min, respectively. Additionally, the tensile strength of gels with varied drug concentrations was measured to analyze the impact of drug load on the mechanical properties of the polymerized bioinks. An Instron (5564 model) was used to measure the failure load of the gels fabricated. The tensile strength (σ) was calculated through Equation (1), where *D* is the gel diameter, *H* is the thickness, and *F* represents the failure load [28].

(1) $$\sigma =\frac{2F}{\pi DH}$$

The inverse of the Ohnesorge number (*Z* value) of the bioinks was calculated to assess the printability of these solutions through inkjet printing piezoelectric nozzles. Equation (2) defines the *Z* value, where a is the radius of the piezoelectric nozzle printing orifice and ρ, γ, and η represent the density, surface tension, and viscosity of the photocurable formula, respectively [29].

(2) $$Z=\frac{{\left(a\rho \gamma \right)}^{1/2}}{\eta }$$

The surface tension was measured with a tensiometer (Kimble Chase 14,818 Tensiometer, Cole-Parmer, Vernon Hills, IL, United States) and calculated by using Equation (3), where h is the distance between menisci of the formulation in the test tube and the one in the capillary tube, r is the radius of the capillary, ρ is the density of the formulation, and g is the acceleration due to gravity [30]. One mL of each bioink was weighted and the mass obtained was divided by the pre-determined volume to calculate the density. The viscosity of the bioinks was measured with a rheometer (Discovery HR-2, TA Instruments, New Castle, DE, USA). A cone and plate geometry (using a 40-mm 2.016°) was utilized for the experiment, with a shear rate ranging from 10 to 100 Hz.

(3) $$\gamma =\frac{1}{2}hr\rho g$$

### 2.4. Scanning Electron Microscopy (SEM)

The morphology of the gels was observed with a NOVA 230 NanoSEM scanning electron microscope. Images of lyophilized hydrophilic gels were taken, as well as cross-sectional images of hydrophobic gels.

### 2.5. Preform Tablet Fabrication and Characterization

The drug-containing bioinks were directly printed into tablet preforms that were fabricated by 3D printing (ProJet 660; 3D Systems, Inc.; Rock Hill, SC, USA). The printing materials consisted of calcium sulfate hemihydrate powder (VisiJet PXL Core; 3D Systems, Inc.; Rock Hill, SC, USA), and a liquid inkjet binder comprised of deionized water containing 5% ethanol and 0.25% Tween 80. The polypill preform tablet was designed with two separate chambers that could each hold up to 250 μL of ink and be assembled into a single tablet. The assembled dimensions were kept below the 22-mm maximum tablet size recommended by the Food and Drug Administration (FDA) [31]. Additionally, each chamber was independently modified to prevent absorption of the respective drug-containing bioink into the preform tablet during printing, as previously reported [23]. Briefly, the chamber holding the hydrophobic formulation was infused with PEG (35 kDa) by submerging the tablet in an acetone solution containing 15% (w/w) PEG for 30 min at 55 °C. Next, the well was brush-coated with Eudagrit^®^ E100 (Evonik, Essen, Germany) (polymethacrylate copolymer) dissolved in acetone at 20% (w/w). The chamber holding the hydrophilic formulation was infused with Eudagrit^®^ E100 dissolved in acetone at 10% (w/w). Surface morphology of the preform tablet was characterized by scanning electron microscopy (NOVA NanoSEM 230, FEI Co., Hillsboro, OR, USA).

### 2.6. Drug Release Kinetics

A piezoelectric dispenser with a nozzle diameter of 80 µm (MJ-ABP-01-080, MicroFab, Plano, TX, USA) was used to assess the droplet formation capability of the engineered bioinks. This dispenser was controlled with a microdispensing system (MD-E-3000, Microdrop, Norderstedt, Germany). The dispensing parameters used were 46 V, a 16-µm pulse width, and a frequency of 2000 Hz. The droplet formation process was captured with an analog camera (JAI CV-S3300), equipped with a lens (Edmund Optics, Barrington, NJ, USA). A light-emitting diode (LED) was connected to the microdispensing system in order to control its strobe delay.

Lisinopril-loaded hydrophilic bioink was dispensed in the bottom part of the polypill (250 µL) and exposed to visible light (120 mW/cm^2^) for 2 min, to induce gelation of the hydrogel precursor solution. Spironolactone-loaded hydrophobic bioink was dispensed into the upper piece of the polypill (125 µL) and exposed to light for 1 min. Following the gelation of the bioinks, the pieces of the polypill were assembled to finalize the pharmaceutical product.

The tablets were placed into uni-cassettes (Tissue-Tek) and immersed into beakers containing 500 mL of dissolution medium (monobasic potassium phosphate 1.053 mM, pH of 2.5), conditioned at 37 °C and stirred at 60 rpm. Aliquots of 1 mL were taken after 0.5, 2, 4, 6, 8, 12, 18, and 24 h. The volume removed was replenished with fresh dissolution medium conditioned at 37 °C. The drug concentration in each aliquot was measured via HPLC (Waters 2690 with a PDA 996 detector). The wavelength used to detect the APIs were 220 nm and 240 nm for lisinopril and spironolactone, respectively.

### 2.7. Statistical Analysis

Statistical analysis was performed with GraphPad Prism software (GraphPad Software, Inc., San Diego, CA, USA). Statistical significance was assessed using single factor ANOVA test with a Tukey post-test and 95% confidence interval.

## 3. Results and Discussion

### 3.1. Bioinks Characterization

**Figure 1:**
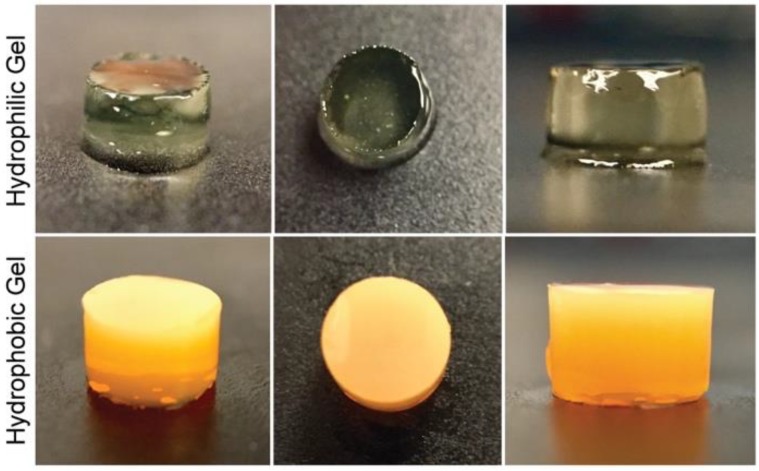
Polymerized drug-loaded bioinks. The upper row shows images of the polymerized hyaluronic acid-based bioink, loaded with lisinopril at a concentration of 40 mg/mL. This bioink has a *W*_HANB_ value of 3% and a *T*_L_ of 2 min. The bottom images show polymerized poly(ethylene glycol) diacrylate (PEGDA) gels with a *W*_PEGDA_ value of 30%, a *W*_EtOH_ value of 20%, and a *T*_L_ of 1.0 min.

**Figure 2:**
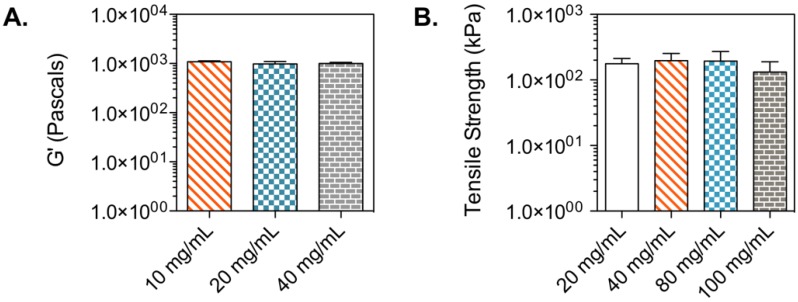
Tensile strength of lisinopril and spironolactone gels. (**A**) Tensile strength of lisinopril tablets with diverse drug concentrations. Forty mg/mL was the maximum solubility achieved for this active pharmaceutical ingredient (API) in this bioink formulation. No statistical difference was observed between samples with varying lisinopril concentrations. (**B**) Tensile strength of spironolactone tablets with diverse drug concentrations. One-hundred mg/mL was the maximum solubility achieved for this API. No statistical difference was observed between samples with varying concentrations.

Hyaluronic acid is a natural glycosaminoglycan found in connective, neural, and epithelial tissues [32,33]. The biocompatibility of hyaluronic acid hydrogels has been assessed in tissue engineering studies, where diverse cell types have been cultured in hydrogels for the formation of tissues and the study of biological processes [34,35,36]. Moreover, hyaluronic acid photocurable formulations can polymerize under quick gelation times (Figure S1). Lisinopril was dissolved in a hyaluronic acid-based hydrophilic bioink at a concentration of 40 mg/mL. The lisinopril formulation was dispensed into a modified syringe (50 µL) and exposed to visible light at an intensity of 120 mW/cm^2^ for 2 min (Figure 1). The storage modulus of the resulting hydrogel was quantified to assess the mechanical properties of the polymerized bioink, due to its viscoelastic property. Additionally, hydrogels with different drug concentrations (20 and 10 mg/mL) were polymerized to study the effect of drug load on the mechanical properties of this hydrophilic material (Figure 2A). The results indicate that this hydrophilic material has a low storage modulus due to its elevated water content and the low *W*_HANB_ (3%) utilized for its fabrication. The elevated water content of the hydrogel allows for the effective diffusion of the hydrophilic API out of the oral dosage form, given the dissolution of the preform tablet designed to disintegrate under acidic conditions similar to the ones found in the stomach. The hydrogels had an average G’ of 1003.86 Pa. Figure 2A indicates that drug load had no influence in the mechanical properties of the hydrogel, where larger drug concentrations had no impact over the G’ of the gels. This result is consonant with the G’ data previously shown [23], where ropinirole-loaded at a concentration of 40 mg/mL had no impact on the mechanical properties of the gel, compared to hydrogels with no drug. However, a decrease in G’ was noticed on hydrogels loaded with ropinirole at a concentration of 80 mg/mL. It can be stated that the G’ remains stable for this lisinopril formulation at concentrations between 0 and 40 mg/mL, the maximum lisinopril concentration achievable in the hyaluronic acid solution.

Spironolactone was chosen as the second model drug in this study due to its high hydrophobicity. The model drug was dissolved in a PEG-based bioink specifically engineered for hydrophobic drugs, at a concentration of 20 mg/mL. Fifty µL of formulation were pipetted into a modified syringe, the solution was exposed to light for 1 min (Figure 1), and the tensile strength of the resulting gel was measured (Figure 2B). Results show an average tensile strength for this material of 176.66 kPa. The typical tensile strength of pharmaceutical tablets is within the range of 1 and 10 MPa [37]. The G’ obtained for this gel is well below this range, and therefore, this material should be used in combination with a preform tablet that provides support and physical stability to the oral dosage form. Furthermore, the effect of drug concentration on the mechanical properties of the gel was studied by measuring the tensile strength of gels containing 100, 80, 40, and 20 mg/mL. Figure 2B shows that drug concentration had no impact on the tensile strength of the material. This contrasts with results previously demonstrated [24], where a decrease in tensile strength was observed with increasing naproxen and ibuprofen concentrations. This divergence is due to the lower *W*_PEGDA_ value used in this formulation (30%). These results indicate that softer materials consisting of photo-polymerized bioinks, such as the hyaluronic acid hydrogel, tend to have a steady mechanical stability when exposed to increasing drug concentrations. Stronger gels can experience substantial differences in mechanical properties (Figure S2).

**Figure 3:**
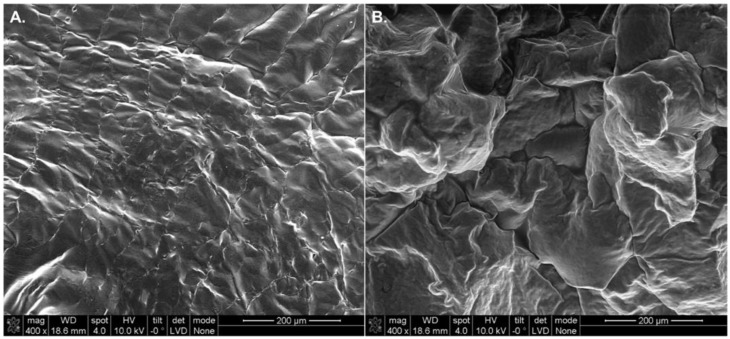
Cross-sectional SEM micrographs of polymerized bioinks loaded with lisinopril and spironolactone. (**A**) Lyophilized lisinopril-loaded hydrogel (40 mg/mL) with a *W*_HANB_ of 3% and a *r*_ratio_ of 0.6. This bioink was exposed to a *T*_L_ of 2 min. (**B**) Polymerized hydrophobic ink loaded with spironolactone (20 mg/mL). This formulation contained a *W*_PEGDA_ of 20% and a *T*_L_ of 1 min.

Scanning electron microscopy imaging was performed on both gels to observe the morphology of the polymerized structures. Figure 3 shows an irregular surface in both gels with small crevices that further facilitate the release of drugs, that otherwise diffuse out of the gels through their polymerized chemical structures. The wrinkles observed in the hydrophilic gel (Figure 3A) are a consequence of the lyophilized/dehydrated nature of the sample tested.

### 3.2. Droplet Formation

**Figure 4:**
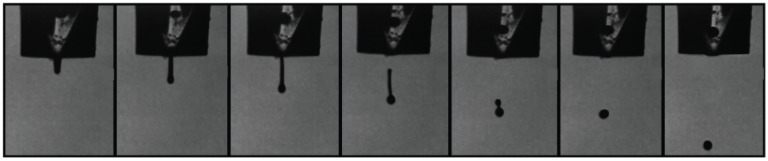
Droplet formation sequence for hyaluronic acid-based bioink with a *W*_PEGDA_ of 3%, a *r*_ratio_ of 0.6, and loaded with lisinopril at a concentration of 40 mg/mL. This formulation had a *Z* value of 6.99, falling within the printable range (4–14).

**Figure 5:**
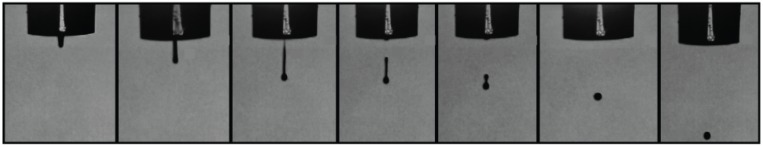
Droplet formation sequence for PEGDA-based bioink with a *W*_PEGDA_ of 30% and loaded with spironolactone at a concentration of 20 mg/mL. This formulation had a *Z* value of 10.52, falling within the printable range (4–14).

To study the droplet formation ability of these bioinks, the inverse of the Ohnesorge number, denominated as *Z* value, was calculated (Equation (3)). This dimensionless number considers the inertia and surface tension forces of a fluid over its viscosity forces to define its droplet formation ability. The orifice radius of the nozzle used for the inkjet printing of the fluid is also a factor taken into consideration within this number. *Z* values between 4 and 14 are considered printable fluids [29]. Values above 14 typically exhibit the formation of satellite droplets, whereas values below four present strong viscous forces. The viscosity, surface tension, and density of the bioinks was quantified, in order to determine their *Z* value. Table 1 shows the results obtained for these parameters at room temperature and the *Z* value for the two bioinks utilized in this study. The hydrophilic bioink experienced a higher viscosity value (9.83 cP), resulting in a lower *Z* value than the hydrophobic formulation. The later one had lower viscosity and surface tension parameters (4.88 cP and 31.41 mN/m, respectively), resulting in a higher *Z* value of 10.52. However, both formulations fell within the defined range for printable fluids as depicted in Figure 4 and Figure 5, where the droplet formation sequence of these bioinks can be observed.

### 3.3. Preform Tablet Characterization

**Figure 6:**
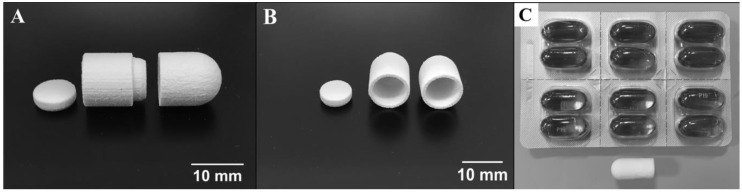
Multi-compartment preform tablet. (**A**) Side view of the three pieces constituting the preform tablet. From left to right, top cap, top compartment, and bottom compartment. These pieces were manufactured by powder-bed 3D printing using as binder DI water with 5% ethanol and 0.25% Tween 80. The powder utilized for their construction was calcium sulfate. Each of the two wells have a 250 µL capacity. (**B**) Front view of the preform tablet fabricated. (**C**) Comparison between the assembled version of the preform tablet and commercially available gel capsules.

**Figure 7:**
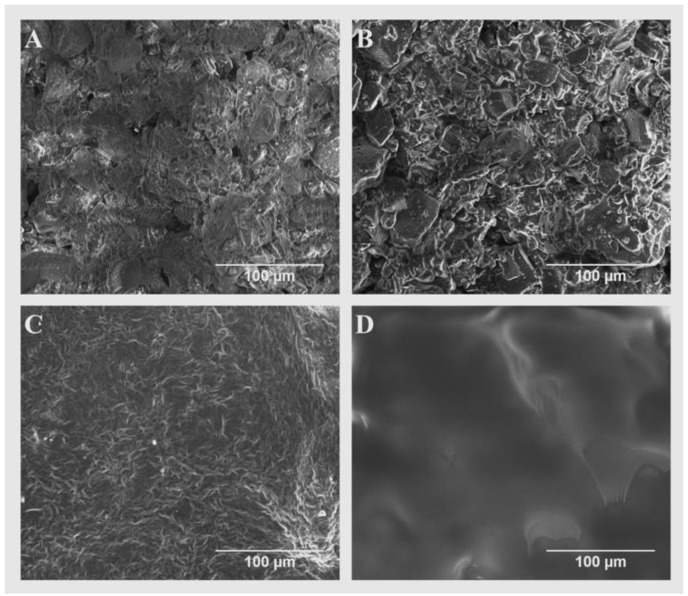
The SEM micrographs of the untreated and coated preform tablet surfaces. (**A**) Surface of untreated preform tablet. (**B**) Bottom compartment infused with a 10% E-100 in acetone solution. (**C**) Top compartment infused with a 15% PEG (35 kDa) in acetone solution. (**D**) Inner section of the top compartment preform tablet, brushed with a 20% E-100 in acetone solution.

The role of the preform tablet was to serve as a vessel for the formulations dispensed prior to their curing, given that their polymerization occurred after a desired amount of formulation was allotted. There was a need to compartmentalize the tablet for each formulation added, since each formulation was cured for different light exposure times to obtain the desired mechanical properties and drug release profiles. Calcium sulfate hemihydrate was clinically used in the preparation of plaster of Paris, which is used for casts that immobilize fractures, and is not used in tablet formulations [38]. Calcium sulfate dihydrate, however, has been commonly used in pharmaceutical applications [39], and the dihydrate is formed when hemihydrate is mixed with water [40]. In this study, water was used as the liquid binder during the fabrication of the perform tablets via 3D printing (Figure 6). Upon contact with the powder, water causes the dissolution of the calcium sulfate hemihydrate and recrystallization of the dihydrate form [41]. The SEM micrographs (Figure 7A) show that pores exist within the uncoated preform tablet wall. These pores negatively impacted the performance of tablets during printing of the bioink. Specifically, the porosity in the preform tablet allowed both the hydrophilic and hydrophobic bioinks to soak into the preform tablet during printing, which not only weakened the preform tablet, but also negates the advantages of using 3D printing for pharmaceutical applications, such as control over drug positioning. To fill the pores, two different coatings were used depending on the ink being used for printing. For the hydrophilic bioink, the preform tablets were soaked in Eudagrit® E100 (Figure 7B). For the hydrophobic bioink, the preform tablets were first soaked in a high-molecular weight PEG (35 kDa) solution. High-molecular weight PEG was selected because it is immiscible with the low-molecular weight PEGDA in the bioink (Figure 7C). Additionally, a thin coating of Eudagrit® E100 was added to the preform tablet well to further inhibit the absorption of the bioink during printing. The SEM micrographs show that the polymeric coating fills the pores of the preform tablet (Figure 7D).

### 3.4. Polypill Dissolution Test

**Figure 8:**
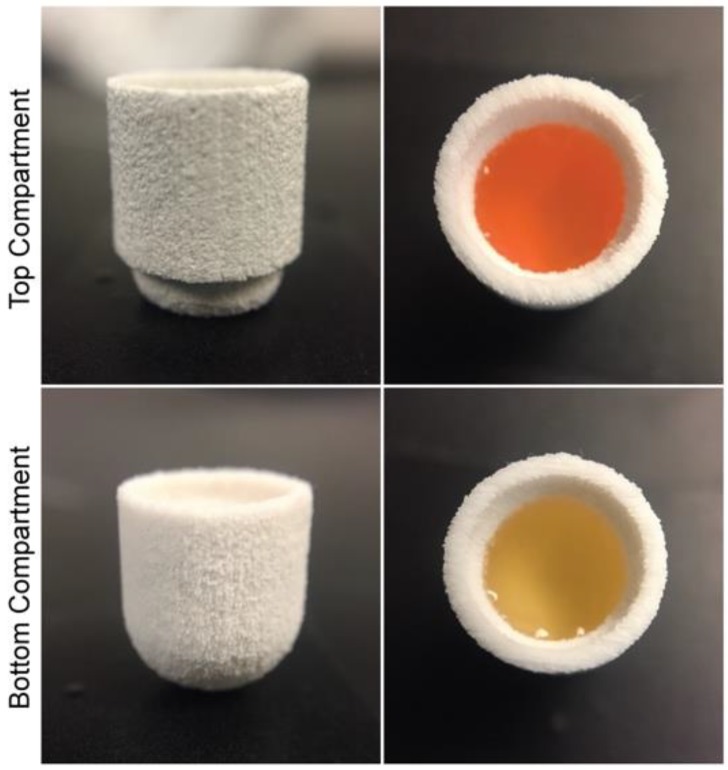
Multi-compartment preform tablet loaded with hydrophilic (bottom) and hydrophobic (top) bioinks.

**Figure 9:**
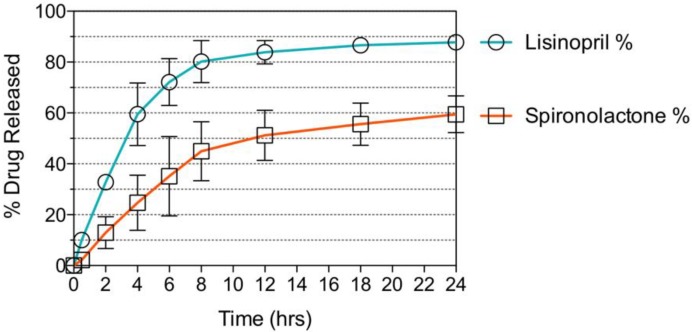
Dissolution test of polypill. A sustained release profile was achieved for both APIs. Around 90% of the lisinopril loaded was release in a period of 24 h, releasing most of the drug within the first 8 h. Above 60% of the spironolactone loaded was released in a period of 24 h.

The hyaluronic acid-based hydrophilic bioink was loaded with lisinopril at a concentration of 40 mg/mL. Two-hundred-and-fifty µL of this formulation were dispensed into the bottom compartment of the preform tablet and further exposed to visible light for 2 min, to induce gelation of the photocurable bioink. Likewise, the PEG-based hydrophobic bioink was loaded with spironolactone at a concentration of 20 mg/mL. One-hundred-and-twenty-five µL of this formulation were loaded into the top compartment of the preform tablet and exposed to visible light for a period of 1 min (Figure 8). Once the bioinks were polymerized, the two compartments were attached, and the small cap was placed to seal the top compartment, completing the oral dosage form. The tablet was immersed in a beaker containing 500 mL of dissolution medium, conditioned at 37 °C and stirred at 60 rpm. Aliquots were taken after 0.5, 2, 4, 6, 8, 12, 18, and 24 h of dissolution and their drug concentration was assessed through HPLC analysis. The results obtained show a dual sustained release of lisinopril and spironolactone in a period of 24 h (Figure 9). The preform tablet dissolved almost in its entirety with the exception of the lower part of the top compartment, completely exposing the gels to the dissolution medium (Figure S3). Lisinopril experienced faster drug release kinetics, when compared to spironolactone. This result can be explained by the differences in the microarchitecture and composition of the gels. The hydrophilic formula has over 90% of water content, facilitating the diffusion of lisinopril, a hydrophilic compound, into the dissolution medium. Moreover, the hydrogel has a *W*_HANB_ of only 3%, allowing small molecules to easily diffuse through the polymerize matrix. The hydrophobic formulation has a higher polymeric content (*W*_PEGDA_ = 30%) resulting in a mesh size of ~11 Å [24]. Naproxen and ibuprofen have a hydrodynamic radius of 3.77 Å and 6.80 Å [42], respectively, and molecular weights of 230.26 Da and 206.29 Da. It can be hypothesized that the hydrodynamic radius of spironolactone is close to the mesh size of the polymerized hydrophobic gel (11 Å), since it has a significantly higher molecular weight than naproxen and ibuprofen, model drugs previously utilized under similar experiment conditions [24]. This would explain the slower release profile observed with spironolactone, since diffusion of the molecule through the gel matrix would be impeded. The use of a higher PEGDA molecular weight for the fabrication of the gel could result in larger mesh sizes and consequently, enhanced drug release kinetics.

The dual release of these APIs for the treatment of hypertension demonstrates the use of inkjet printing for the fabrication of combination therapies. Moreover, it shows that the therapy could contain both hydrophilic and hydrophobic compounds. This technology would be especially applicable towards drugs that achieve their pharmacological effect at low dosages and could be targeted towards the development of oral dosage forms for children who require small dosages not always commercially available. Moreover, children experience drastic changes in metabolism that affect the dosage needed to achieve a given target pharmacological effect.

## 5. Conclusions

In this study, a combination therapy oral dosage form for the treatment of hypertension was designed. The polypill featured the use of a hyaluronic acid photocurable hydrophilic bioink and a PEG photocurable hydrophobic bioink, loaded with lisinopril and spironolactone, respectively. A preform tablet with two compartments able to hold these bioinks was designed and manufactured through binder jetting 3D printing. The formulations were dispensed through a piezoelectric nozzle designed for material jetting and subsequently polymerized through exposure to visible light. The preform parts were assembled and a dissolution study was carried out, where a dual sustained release of the drugs lisinopril and spironolactone was observed over a period of 24 h. This study shows the feasibility of 3D printing photocurable formulations to manufacture combination therapy oral dosage forms that incorporate both hydrophilic and hydrophobic APIs, with a special application towards drugs that achieve their pharmacological effect at low dosages.

## Figures and Tables

**Table 1:** Physical properties and *Z* value of formulated bioinks.

Bioink	r	*P* (kg/m^3^)	γ	η (mPa·s)	*Z*
Hydrophilic	0.08	1022.27	57.76	9.83	6.99
Hydrophobic	0.08	1048.00	31.41	4.88	10.52

## Supplementary Materials

The following are available online at http://www.mdpi.com/2073-4360/10/12/1372/s1, Figure S1: Gelation kinetics obtained through in situ photorheology of the hydrophilic bioink, Figure S2: Tensile Strength of PEGDA-based (*W*_PEGDA_ = 100%) gels loaded with varying concentrations of spironolactone, Figure S3: Exposed gels after 24 h of dissolution time.
