# Supplementary material for: Photocurable Bioinks for the 3D Pharming of Combination Therapies
Source: Polymers (Basel). 2018 Dec 11;10(12):1372. doi: 10.3390/polym10121372 (PMC6401852; doi:10.3390/polym10121372)
Supplement: Supplementary file 1 [file polymers-10-01372-s001.pdf]

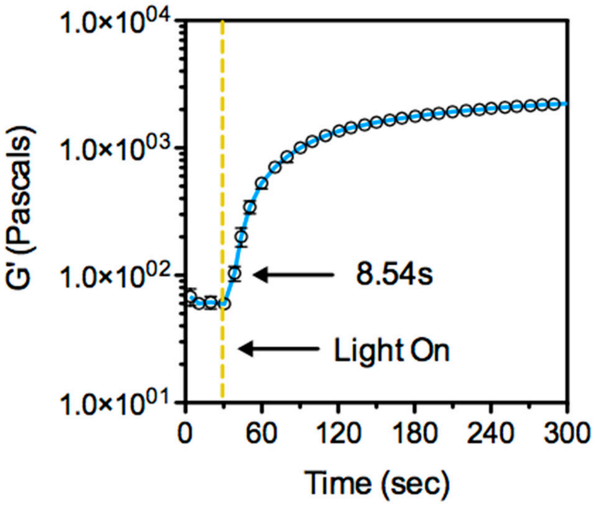

Figure S1. Gelation kinetics obtained through in-situ photorheology of the hydrophilic bioink. The gelation kinetics were assessed through in-situ photorheology in a rheometer, at a constant strain of 1% and an angular frequency of 10 rad/sec. In this test, the storage modulus (G') of the bioinks was measured for 30 s without light exposure. Immediately after this time, a visible light source was turned on and the G' was measured for another 270 s. Results show that the formulation achieves a G' higher than 100 Pa in 8.54 s.

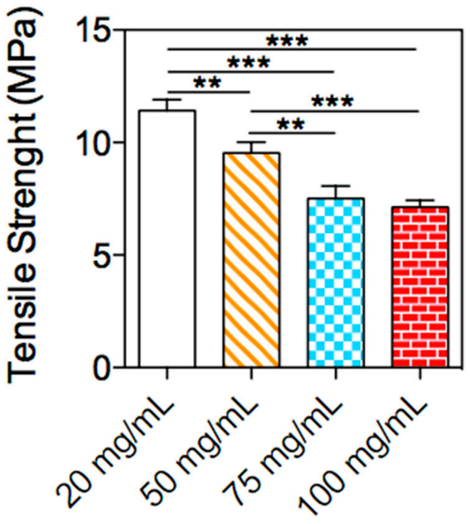

Figure S2. Tensile Strength of PEGDA based (W_{PEGDA} = 100%) gels loaded with varying concentrations of Spironolactone. These gels were exposed to light for a period of 3 min. We can see that light exposure time has a direct effect on tensile strength, since significant differences were observed under these conditions, diverging from results shown in Figure 2B.

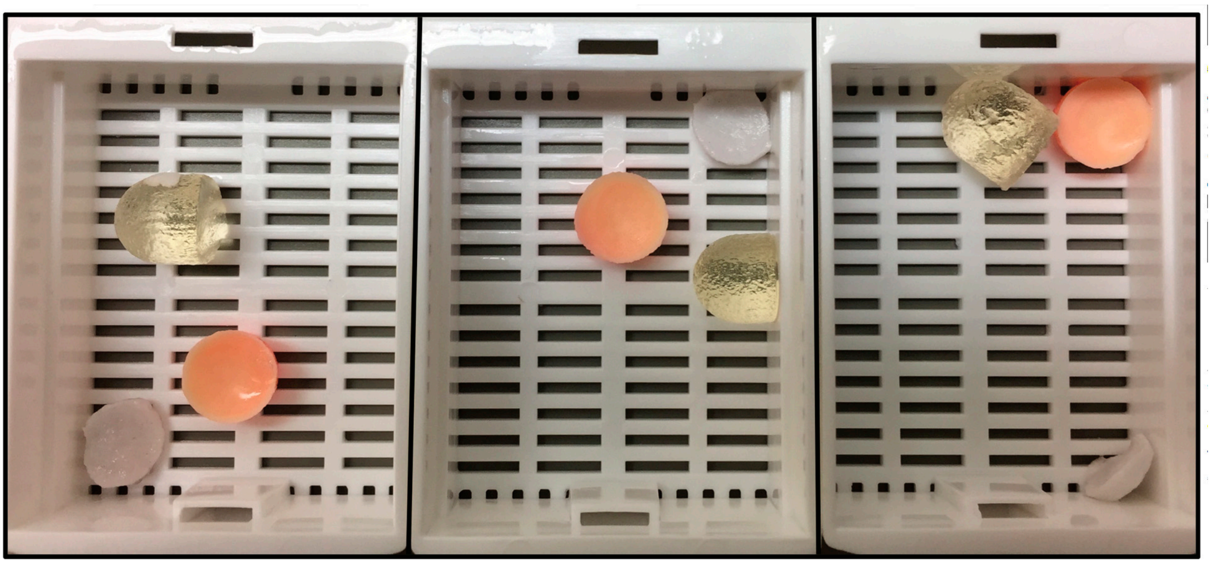

Figure S3. Exposed gels after 24 h of dissolution time. It can be observed that most of the preform tablet is dissolved after the dissolution process.
